# Derivation and Identification of Motor Neurons from Human Urine-Derived Induced Pluripotent Stem Cells

**DOI:** 10.1155/2018/3628578

**Published:** 2018-01-24

**Authors:** Huan Yi, Bingbing Xie, Ben Liu, Xuan Wang, Li Xu, Jia Liu, Min Li, Xiufeng Zhong, Fuhua Peng

**Affiliations:** ^1^Department of Neurology, The Third Affiliated Hospital of Sun Yat-sen University, Guangzhou, Guangdong 510630, China; ^2^State Key Laboratory of Ophthalmology, Zhongshan Ophthalmic Center, Sun Yat-sen University, Guangzhou, Guangdong 510600, China; ^3^Department of Dermatology, The Third Affiliated Hospital of Sun Yat-sen University, Guangzhou, Guangdong 510630, China

## Abstract

Induced pluripotent stem cells (iPSCs) have provided new opportunities for motor neuron disease (MND) modeling, drug screening, and cellular therapeutic development. Among the various types of iPSCs, urine-derived iPSCs have become a promising source of stem cells because they can be safely and noninvasively isolated and easily reprogrammed. Here, for the first time, we differentiated urine-derived iPSCs (urine-iPSCs) into motor neurons (MNs) and compared the capacity of urine-iPSCs and cord-blood-derived iPSCs (B-iPSCs) to differentiate into MNs. With the use of small molecules, mature MNs were generated from urine-iPSCs as early as 26 days in culture. Furthermore, in coculture with muscle cells, MNs projected long axons and formed neuromuscular junctions (NMJs). Immunofluorescence and PCR confirmed the expression levels of both MN and NMJ markers. The comparison of the ratios of positive labeling for MN markers between urine-iPSCs and B-iPSCs demonstrated that the differentiation potentials of these cells were not significantly different. The abovementioned results indicate that urine-iPSCs are a new, promising source of stem cells for MND modeling and further cellular therapeutic development.

## 1. Introduction

Motor neuron diseases (MNDs) selectively affect motor neurons (MNs), which project axons to muscles and control voluntary actions. Patients with MNDs may present a range of symptoms, such as muscular weakness, atrophy, and hyperreflexia, which ultimately lead to death [1]. No effective treatments are available for MND. Thus, pluripotent stem cells (PSCs) have become an important tool for the study of MND and represent a promising therapeutic approach [2]. Among the variety of stem cells, induced pluripotent stem cells (iPSCs), which are reprogrammed from adult somatic cells, are very advantageous for MND modeling, drug discovery, and individual therapeutic transplantation, without ethical concerns [3]; all of these efforts have made substantial progress toward understanding MND. To date, multiple types of somatic cells have been reprogrammed into iPSCs, including widely used skin fibroblasts and peripheral blood cells, which have the potential to differentiate into MNs [4]. Compared with these cells, urinary cells provide a convenient, cost-effective, and noninvasive source of cells that can be obtained and reprogrammed into iPSCs [5]. However, it is unclear whether urine-derived human iPSCs have the capacity to differentiate into MNs. In this study, we rapidly and efficiently induced the differentiation of urine-derived iPSCs (urine-iPSCs) into MNs. Immunofluorescence and PCR confirmed the expression levels of neural markers at every stage of induction and MN-specific markers of cells derived from urine-iPSCs. We also demonstrated the functional capacity of MNs to form NMJs in cocultures of urine-derived MNs and muscle cells.

## 2. Materials and Methods

### 2.1. iPSC Cultures

The human iPSC lines used in this study included two urine cell-derived iPSC lines, UE017 and UC005, obtained from the Chinese Academy of Sciences, Guangzhou Institute of Biomedicine and Health [6]. As a control, a cord blood-derived iPSC (B-iPSC) line, which was purchased from Gibco (USA, catalog A18945), was used in this study. All iPSCs were cultured on Matrigel-coated plates with mTeSR I (STEMCELL Technologies, Canada), which was changed daily. Immunofluorescence was performed to identify the expression profiles of pluripotency markers.

### 2.2. Differentiation of iPSCs into MNs

**Figure 1:**
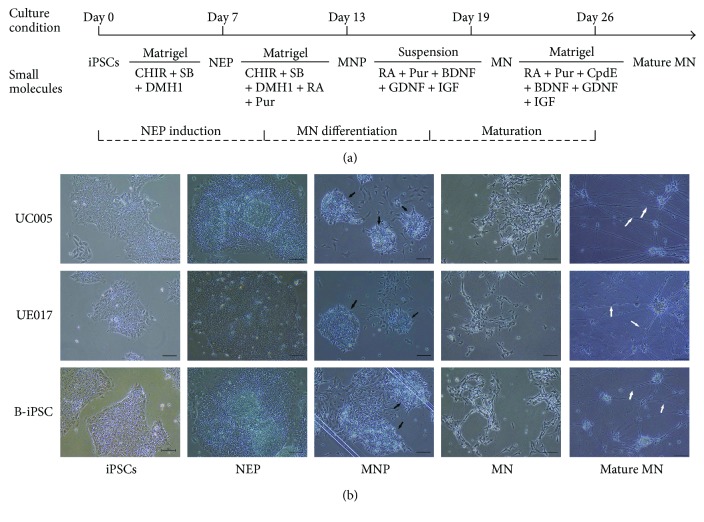
(a) Time course and small-molecule cocktail for the differentiation of iPSCs into mature MNs. Following exposure to CHIR99021, SB431542, and DMH1 for 6 days, the iPSCs differentiated into NEPs. Over 6 days, with the addition of RA and Pur, the cells differentiated into MNPs. During the last 2 weeks of differentiation, in the presence of RA, Pur, and neurotrophic factors, the MNPs finally differentiated into mature MNs. (b) Morphology of the cells derived from the three iPSC lines at every differentiation stage. Before the differentiation process, the three iPSC lines exhibited uniform, undifferentiated morphology. After 6 days of induction, the cells exhibited inconsistent size and shape and aggregated centrally (D7, NEPs). With an additional 6 days of differentiation, cell morphology changed quickly, and the cells started to gather centrally to form rosettes (D13, MNPs, black arrow). On day 19, the differentiated MNs began projecting axons. The MNs matured gradually, and their axons elongated over time (D26, white arrow). The scale bar is 100 *μ*m.

Du's protocol for the differentiation of iPSCs into MNs was used with slight modification (Figure 1(a)) [7]. For MN generation, undifferentiated iPSCs were dissociated with 5 *μ*M EDTA (Invitrogen) for 5 min and then passaged in Matrigel-coated plates in 1 : 6. The following day, the stem cell medium mTeSR was replaced with neural differentiation medium (NDM) with the addition of 3 *μ*M CHIR99021 (CHIR, Sigma), 2 *μ*M DMH1 (Sigma), and 2 *μ*M SB341542 (SB, Sigma). NDM includes Dulbecco's Modified Eagle Medium (DMEM/F12), Neurobasal Medium at a 1 : 1 concentration, 0.5 × N2, 0.5 × B27, 0.1 mM ascorbic acid (Sigma), 1 × Glutamax, and 1 × antibiotic-antimycotic (all others from Invitrogen). The medium was changed every other day. IPSCs maintained under these conditions for 6 days differentiated into neuroepithelial progenitors (NEPs). On day 7, the NEPs were treated with dispase (1 mg/ml) for 5 min and then gently resuspended with NDM, including 1 *μ*M CHIR, 2 *μ*M DMH1, 2 *μ*M SB, 0.1 *μ*M retinoic acid (RA, Sigma), and 0.5 *μ*M purmorphamine (Pur, Sigma), and were plated on Matrigel-coated plates at a 1 : 3 concentration. The culture medium was changed every other day for 6 days, and on day 12, the NEPs differentiated into motor neuron progenitors (MNPs), which aggregated in rosettes. To induce MNPs into MNs, the rosettes were lifted with dispase (1 mg/ml) and then cultured in suspension in NDM with 0.5 *μ*M RA, 0.1 *μ*M Pur, 10 ng/ml brain-derived neurotrophic factor (BDNF, PeproTech), 10 ng/ml glial cell line-derived neurotrophic factor (GDNF, PeproTech), and 10 ng/ml insulin-like growth factor (IGF, PeproTech) for 6 days. On day 19, HB9-positive MN spheres formed. To induce further maturation, these MN spheres were dissociated with accutase (Invitrogen) into single neurons and cultured adherently with NDM, including 0.5 *μ*M RA, 0.1 *μ*M Pur, 0.1 *μ*M Compound E (Cpd E, Calbiochem), and the abovementioned neurotrophic factors, for more than 7 days. Then, MNs differentiated into mature MNs.

### 2.3. Coculture of iPSC-Derived MNs and C2C12 Cells

C2C12 cells, a mouse myoblast cell line, were obtained from the China Center for Type Culture Collection and cultured in growth medium (DMEM/F12 supplemented with 10% fetal bovine serum (FBS)) until the cells reached 60–70% confluence. Then, the medium was changed to differentiation medium (DMEM/F12 with 2% horse serum (HS)), and myoblasts cultured in this medium for 4 days differentiated into skeletal muscle cells. On day 19, UC005-derived MN spheres were dissociated into single MNs, the MNs were added to each dish of muscle cells, and the medium was replaced with NDM, which was supplemented with RA, Pur, BDNF, GDNF, and IGF. Within 1-2 days, the MNs projected axons, and after more than 1 week in culture, the MNs formed NMJs with the muscle cells.

### 2.4. Immunofluorescence

The cultured cells were placed on 12 mm cover slips, fixed in 4% paraformaldehyde (PFA; Sigma) for 10 min at room temperature, washed three times with phosphate-buffered saline (PBS, Geno, China), and treated with a permeabilizing and blocking buffer (10% donkey serum, 0.225% Triton X-100) for 1 hour at room temperature. Then, the cells were incubated with the following primary antibodies: OCT4 (1 : 200, Abcam), Nanog (1 : 250, Abcam), SSEA4 (1 : 250, Abcam), TRA-1-60 (1 : 300, Abcam), SOX1 (1 : 300, Boster), SOX2 (1 : 300, Boster), Nestin (1 : 300, Abcam), Olig2 (1 : 500, Millipore), Pax6 (1 : 100, DSHB), HB9 (1 : 50, DSHB), Islet1 (1 : 250, Abcam), ChAT (1 : 100, Millipore), and TuJ1 (1 : 250, Abcam). All antibodies were diluted in antibody dilution buffer (2% donkey serum, 0.05%Triton X-100), and the cells were incubated with the antibodies overnight at 4°C. After three washing steps, the cells were incubated for 1 hour at room temperature with secondary antibodies: Alexa Fluor (488 or 555) donkey anti-mouse, donkey anti-rabbit, and donkey anti-goat. For the detection of AChR, the cocultured cells were incubated with Alexa Fluor 555-conjugated *α*-BTX (1 *μ*g/ml, Invitrogen) for 1 hour at 37°C before fixation. The cells were then washed 2 times in PBS and fixed with 4% PFA. All cell samples were observed using an Olympus fluorescence microscope.

### 2.5. RNA Isolation and RT-PCR

The mRNA expression levels of neuronal markers were analyzed using reverse transcriptase polymerase chain reaction (RT-PCR). TRIzol (Sigma), chloroform, isopropanol, and DNase I were used for total RNA extraction. Then, 0.8 *μ*g isolated RNA was reverse-transcribed to cDNA using the PrimeScript™ RT Master Mix (TAKARA, Kyoto, Japan). The PCRs contained 1.5 *μ*l cDNA, 10 *μ*l Ex Taq (TAKARA), 0.8 *μ*l primers (Table 1), and 7.7 *μ*l nuclease-free H_2_O. The PCR conditions consisted of 98°C for 2 min, followed by 40 cycles of 98°C for 10 sec, 65°C for 30 sec, and 72°C for 30 sec. For the PCRs, GAPDH was chosen as a housekeeping gene.

### 2.6. Statistical Analysis

GraphPad Prism 7 Software (GraphPad Software) was used for statistical analysis. The presented results are from three independent experiments. Statistical significance was determined with the *t*-test, and the results are presented as the means ± standard errors of the mean. The *P* values ^∗^*P* < 0.01, ^∗∗^*P* < 0.001, and ^∗∗∗^*P* < 0.0001 were considered significant.

## 3. Results

### 3.1. Characterization of iPSCs

**Figure 2:**
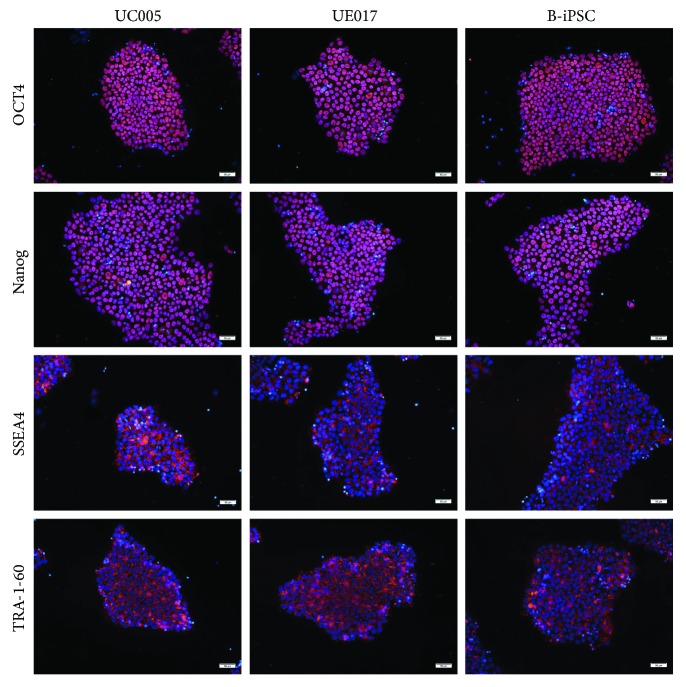
Pluripotency marker expression in iPSCs. Three iPSC lines, B-iPSC, UE017, and UC005, were positive for pluripotency markers, including OCT4, Nanog, SSEA4, and TRA-1-60. Cell nuclei were counterstained with 4′,6-diamidino-2-phenylindole (DAPI). The scale bar is 50 *μ*m.

Before initiation of the differentiation process, we first analyzed the pluripotent properties and purity of the iPSCs. Both urine-iPSCs and B-iPSCs exhibited uniform undifferentiated morphology, including a round shape, large nucleoli, scant cytoplasm, and organized colonies, similar to the features of embryonic stem cells (ESCs) (Figure 1) [8]. All iPSC lines mentioned above, including UC005, UE017, and B-iPSC, were confirmed by immunofluorescence staining of the pluripotency markers, including OCT4, Nanog, TRA-1-60, and SSEA4 (Figure 2) [9].

### 3.2. Induction of NEPs by Small Molecules

**Figure 3:**
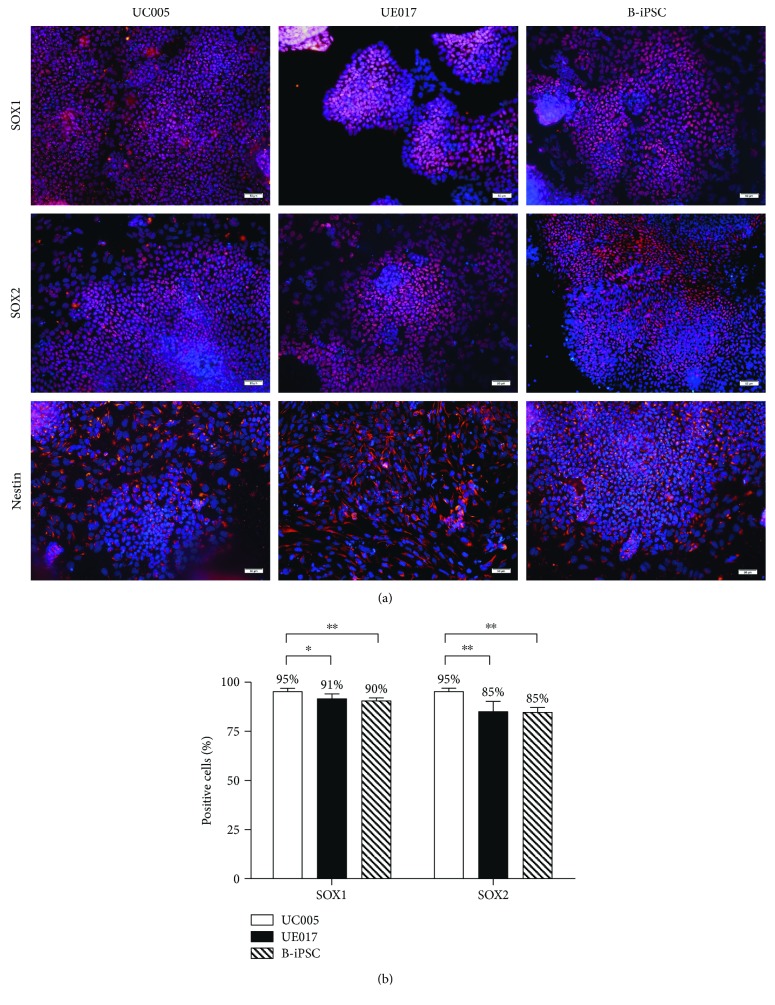
NEPs derived from urine-iPSCs and B-iPSCs express neural progenitor markers. (a) Immunocytochemical staining of neural progenitor markers. NEPs derived from all three iPSC lines, B-iPSC, UC005, and UE017, were positive for the neural precursor markers, SOX1, SOX2, and Nestin. Cell nuclei were counterstained with 4′,6-diamidino-2-phenylindole (DAPI). The scale bar is 50 *μ*m. (b) The ratio of positive labeling for SOX1 and SOX2 in NEPs after 7 days of induction. NEPs from UC005 expressed higher levels of neural precursor markers. The ratio of positive labeling (%) = (number of positive cells/total number) × 100%. The results from three independent experiments are presented as the means ± standard errors of the mean (SEM). ^∗∗^*P* < 0.001 and ^∗^*P* < 0.01 according to Student's *t*-test.

**Figure 4:**
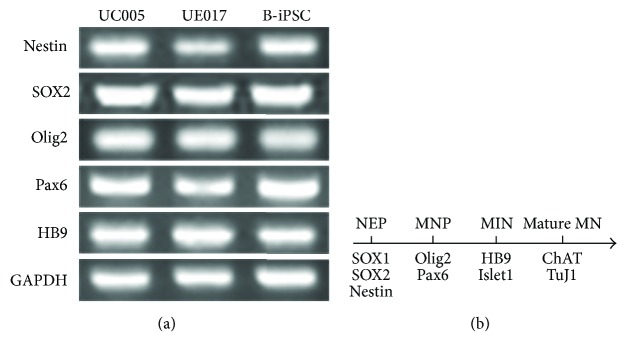
(a) PCR analysis of the mRNA expression levels of neural cell markers at every differentiation stage. After 7 days of differentiation, both B-NEPs and urine-NEPs expressed the neural markers, Nestin and SOX2. After an additional 6 days of induction, cells derived from all three iPSC lines expressed the MNP markers, Olig2 and Pax6. As early as day 19 of induction, MNs from B-iPSCs and urine-iPSCs expressed the MN-specific marker, HB9. These mRNA expression results were consistent with the results of protein expression, as shown by immunofluorescence. (b) Stages of differentiation of MNs from iPSCs and markers commonly used for their characterization.

To demonstrate the potential of urine-derived iPSCs to differentiate into MNs, we induced both B-iPSCs and urine-iPSCs (UE017, UC005) into MNs (Figure 1). The specification of MNs is determined by the following steps: neutralization, caudalization, and ventralization [10]. The first step, neutralization, was activated by the combined inhibition of the bone morphogenetic protein (BMP) and transforming growth factor beta (TGF*β*) signaling pathways [11]. The GSK-3*β* inhibitor promotes neural progenitor proliferation by stimulating the canonical Wnt signaling pathway, which contributes to the maintenance of neural precursors [12]. Based on these preliminary studies [7, 13], when the confluence of the iPSC colonies reached 70–80%, we dissociated the iPSCs, cultured them adherently in new Matrigel-coated plates, and then replaced the medium with NDM, which included DMH1 and SB431542 (inhibitors of BMP/TGF*β*) and CHIR99021 (inhibitor of GSK-3*β*), to evoke neural induction. The iPSCs maintained under these neutralized conditions for 6 days differentiated into NEPs that exhibited obvious changes in morphology. The irregular, polygon-shaped cells aggregated centrally, and the peripheral cells were larger than the central cells (Figure 1(b)). Immunocytochemical staining revealed positive labeling for NEPs through the expression of neural progenitor markers SOX1, SOX2, and Nestin (Figure 3(a)) [14]. The majority of cells derived from all three iPSC lines expressed these pan-neural markers. By counting the positive cells, we determined that the positive ratio of NEPs derived from UC005 (UC005-NEP) with SOX1 and SOX2 immunolabeling was significantly higher than that of B-NEP and UE017-NEP (Figure 3(b)). The RT-PCR also confirmed the expression of the neural markers SOX2 and Nestin (Figure 4).

### 3.3. Efficient Neural Induction and MN Generation

**Figure 5:**
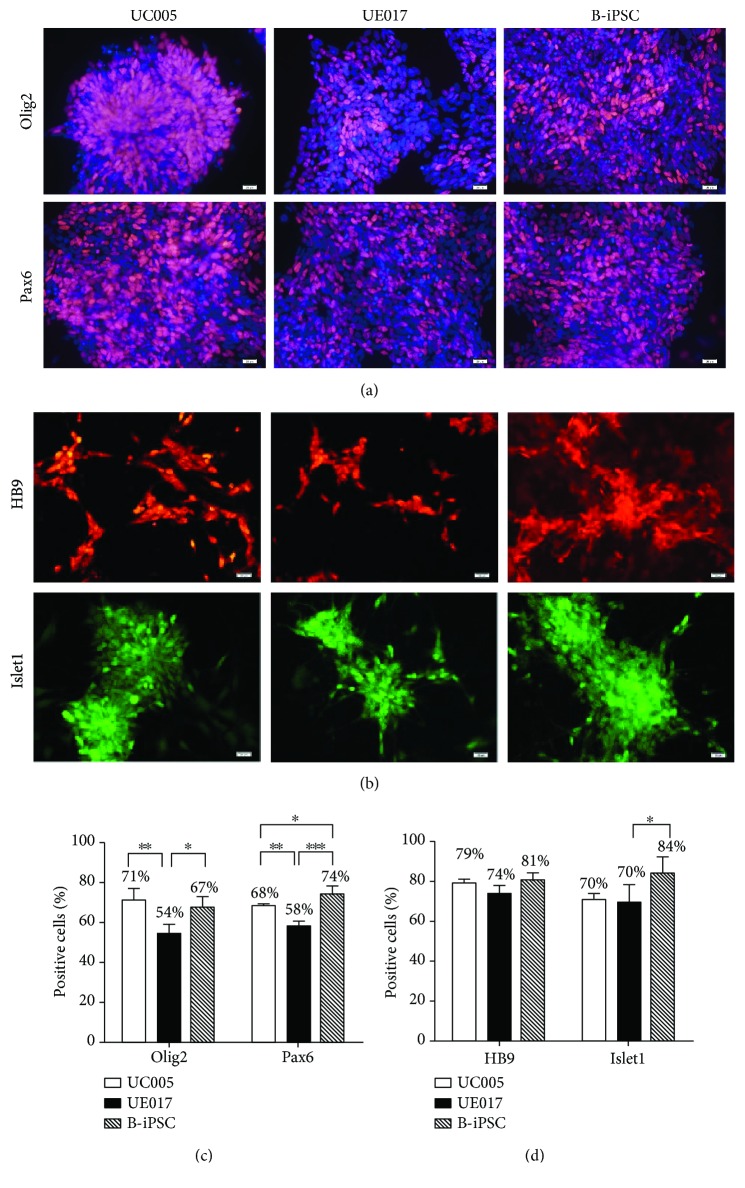
Immunocytochemical staining and ratios of positive labeling of the MNPs and MNs. (a) After 13 days of induction, cells derived from all three iPSC lines, UC005, UE017, and B-iPSC, were positive for the MNP markers, Olig2 and Pax6. Cell nuclei were counterstained with 4′,6-diamidino-2-phenylindole (DAPI). The scale bar is 50 *μ*m. (b) After 19 days of induction, the cells derived from all three iPSC lines, UC005, UE017, and B-iPSC, were positive for the MN markers, HB9 and Islet1. Cell nuclei were counterstained with 4′,6-diamidino-2-phenylindole (DAPI). The scale bar is 50 *μ*m. (c) Cells from the UC005 and B-iPSC lines expressed higher levels of the MNP markers than the cells from the UE017 line. (d) The ratios of positive labeling for HB9 and Islet1 in MNPs after 19 days of induction. The ratios of positive labeling for HB9 were 79%, 74%, and 81% in MNs derived from UC005, UE017, and B-iPSC, respectively, but the difference was not statistically significant. A total of 84% of MNs from B-iPSC expressed Islet1, which was significantly higher than that of MNs from UC005 and UE017. The ratio of positive labeling (%) = (number of positive cells/total number) × 100%. ^∗∗∗^*P* < 0.0001, ^∗∗^*P* < 0.001, and ^∗^*P* < 0.01 according to Student's *t*-test.

The further induction of NEPs into MNPs refers to caudalization and ventralization, which are activated by the Sonic hedgehog (Shh) signaling pathway and RA [10]. The NEPs that were exposed to 0.1 *μ*M RA, 0.5 *μ*M Pur (an activator of the Shh signaling pathway), DMH1, SB, and CHIR for 6 days exhibited rapid changes in morphology. At this stage, the induced cells gathered centrally and formed rosettes (Figure 1(b)), neural tube-like structures with differentiation potential toward the central nervous system (CNS) and peripheral nervous system (PNS) fates [15]. The cells in the rosette were positive for the MNP markers [16], Olig2 and Pax6, but the peripheral cells bordering the rosette were negative (Figure 5(a)). Immunocytochemical staining and RT-PCR verified the expression of the MNP markers in both urine-iPSC- and B-iPSC-induced MNPs (Figures 4 and 5(a)). When we compared the ratios of cells positive for the MNP markers among the cells derived from all three iPSC lines, the expression levels of both Olig2 and Pax6 in cells derived from B-iPSCs (B-MNPs) and UC005 (UC005-MNPs) were significantly higher than those in cells from UE017 (UE017-MNPs); furthermore, the Pax6 expression levels in B-MNPs were significantly higher than those in UC005-MNPs (Figure 5(c)).

### 3.4. MN Specification and Maturation

**Figure 6:**
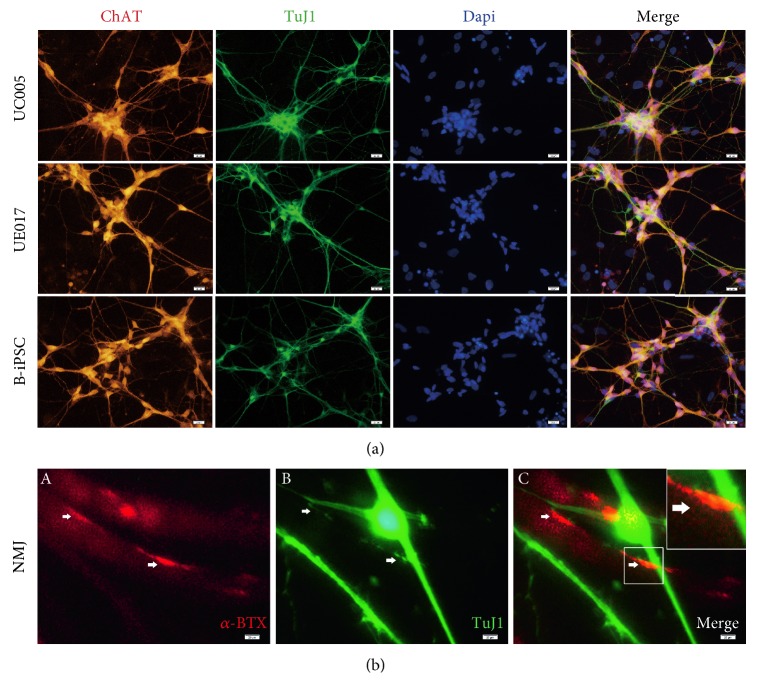
Mature and functional MNs were generated after 26 days of differentiation. (a) Immunocytochemical staining of mature MNs. Under inductive conditions for more than 26 days, cells derived from all three iPSC lines, B-iPS, UE017, and UC005, expressed the mature MN marker choline acetyltransferase (ChAT). They also expressed the neuronal marker TuJ1. Cell nuclei were counterstained with 4′,6-diamidino-2-phenylindole (DAPI). (b) Cocultured muscle cells and MNs form neuromuscular junctions. Immunofluorescence staining shows *α*-bungarotoxin- (BTX-) positive sites overlaid with TuJ1 labeling in the UC005 MNs (A, B, C). Cell nuclei were counterstained with 4′,6-diamidino-2-phenylindole (DAPI). The scale bar is 50 *μ*m.

To induce MNPs into functional MNs, we cultured MNPs in suspension with decreased Pur (0.1 *μ*M) and increased RA (0.5 *μ*M) [7] coupled with BDNF, GDNF, and IGF (each 10 ng/ml). After 6 days under the maturation conditions, MN floating spheres formed, as evidenced by the expression of HB9, an immature motor neuron marker [17], and LIM-homeodomain transcription factor Islet1 [18] (Figure 5(b)). The ratio of positive labeling for HB9 in B-iPSC-derived MNs (B-MNs) was higher than that in MNs derived from UC005 (UC005-MNs) and UE017 (UE017-MNs), but the difference was not statistically significant (Figure 5(d)). We tried to culture cells adherently in this stage to avoid damaging the dissociated spheres in the next stage; however, the ratio of positive labeling decreased. Then, we dissociated these MN spheres into single cells and cultured them on Matrigel with Pur, RA, and Cpd E (an inhibitor of NOTCH). The next day, the adherent urine-MNs and B-MNs projected axons. When the MNs were cultured under these conditions for more than 7 days, the MNs matured even further and projected long axons; axonal elongation occurred gradually over time (Figure 1(b)). MN maturation was evidenced by immunofluorescence staining of the neural marker TuJ1 and the mature MN-specific marker ChAT (Figure 6(a)) [19, 20]. RT-PCR further verified the mRNA expression of the MN-specific marker HB9 in both urine-MNs and B-MNs, consistent with immunofluorescence observations (Figure 4).

### 3.5. NMJs Formed between Urine-MNs and Muscle Cells

On day 19 of neural differentiation, dissociated UC005-MNs were added to muscle culture cell dishes, and after 5 days of coculturing, muscle cell contraction was observed with a brightfield microscope. Within additional 2-3 days, immunofluorescence staining revealed positive labeling for the acetylcholine receptor (AChR) marker, *α*-bungarotoxin (*α*-BTX), on the surface of the muscle cells [21] and colocalization of *α*-BTX and TuJ1 at sites on the axons of the UC005-MNs (Figure 6(b)), which indicated accumulation of AChR and the formation of NMJs between urine-MNs and muscle cells at these sites [22].

## 4. Discussion

Directed differentiation of stem cells into MNs holds a great promise for the in vitro modeling of neurodegenerative diseases and cellular replacement therapies [23]. Several protocols for MN differentiation have been reported [7, 24–26]. Among these protocols, Du's protocol yielded a highly pure population of MNs that differentiated from iPSCs. To date, different sources of iPSCs, including skin fibroblasts and blood cells, have been used in these MN differentiation protocols [27, 28]. Compared to skin fibroblasts and blood cells, urinary cells are safer and more efficient for isolation and reprogramming [4]. In this study, for the first time, we demonstrate the potential of urine-iPSCs to differentiate into MNs. We further investigated the capacities of blood cell-derived iPSCs and urine-iPSCs to differentiate into MNs by comparing the expression levels of neural markers.

We used Du's differentiation protocol [7], with slight modification, to induce MNPs to differentiate into mature MNs. To promote steady differentiation, the cells were treated with RA, Pur, Cpd E, and neurotrophic factors, which were not consistently added in Du's protocol. Before the differentiation of iPSCs into MNs, urine-iPSCs (UC005, UE017) exhibited characteristic properties of PSCs, which was confirmed by immunofluorescence staining of pluripotency markers. Then, activation induced by BMP and TGF*β* combined with inhibition of GSK-3*β* initiated the neutralization of iPSCs. After 7 days of induction, both B-iPSCs and urine-iPSCs differentiated into NEPs. The ratios of positive labeling for the neural precursor markers in NEPs derived from the three iPSC lines were all greater than 85%, and the ratio of the UC005-NEPs was slightly higher than those of the other two cell lines. During the subsequent 6 days, the presence of RA and Pur resulted in rapid changes in morphology, formation of rosettes, and upregulation of the MNP markers Olig2 and Pax6. The expression levels of the MNP markers in the UC005-MNPs and B-MNPs were significantly higher than those in the UE017-MNPs. With further differentiation in suspension, cells derived from all three iPSC lines congregated and formed MN spheres, which expressed the MN markers HB9 and Islet1. The rates of positive labeling for the precursor neural markers and MNP markers were significantly different among the cells from UC005, UE017, and B-iPSC, but the rates of positive labeling for the MN marker, HB9, were not statistically significant among the cells. For the further MN maturation, the combined actions of RA, Pur, and Cpd E resulted in the projection of long axons and expression of the mature MN marker, ChAT, in MNs derived from all three iPSC lines. When cocultured with muscle cells, urine-iPSC-derived MNs exhibited functional properties, including the projection of axons toward muscle cells, induction of muscle contractions, and NMJ formation. To further confirm our immunocytochemical observations, we used RT-PCR to analyze the mRNA expression levels of neuronal- and MN-specific markers. In all three iPSC lines, we detected expression of neuronal markers (SOX1, Nestin, Pax6, and Olig2) and the MN-specific marker, HB9.

In conclusion, the expression profiles of neural cell markers, as measured by immunocytochemistry and RT-PCR, demonstrated that urine-iPSCs were able to successfully differentiate into MNs. The formation of NMJs between MNs and muscle cells further indicated the functional properties of urine-MNs. In addition, the comparison of the ratios of positive labeling for MN markers demonstrated that the capacity of urine-iPSCs and B-iPSCs to differentiate into MNs is not significantly different. However, there are some important aspects that we did not investigate, such as the electrophysiological properties of induced MNs in vitro [29]. Furthermore, transplantation of MNs into an MND animal model is necessary for future studies of cell regenerative medicine [30]. These two aspects mentioned above will be explored in our future research. In this study, we demonstrated the feasibility of differentiating urine-derived iPSCs into MNs. Since urine-derived iPSCs can be easily accessed in a noninvasive manner, urine-derived iPSCs provide a novel platform for disease modeling, drug screening, and cellular therapy for MND.

## Figures and Tables

**Table 1:** Primers used for reverse transcription polymerase chain reaction (RT-PCR).

Gene	Primer sequence (5′-3′)	Annealing (°C)
Nestin	F AGAAACAGGGCCTACAGAGC; R GAGGGAAGTCTTGGAGCCAC	65°C
SOX2	F CCCCCGGCGGCAATAGCA; R TCGGCGCCGGGGAGATACAT	60°C
Olig2	F CCCTAAAGGTGCGGATGCTT; R CTGGATGCGATTTGAGGAGC	65°C
Pax6	F CGGAGTGAATCA GCTCGGTG; R CCGCTTATACTGGGCTATTTTGC	60°C
HB9	F AGCACCAGTTCAAGCTCAACA; R ACCAAATCTTCACCTGGGTCTC	65°C
GAPDH	F ACCACAGTCCATGCCATCAC; R TCCACCACC CTGTTGCTGTA	60°C
